# Kinematic Analysis of the Upper Limb Motor Strategies in Stroke Patients as a Tool towards Advanced Neurorehabilitation Strategies: A Preliminary Study

**DOI:** 10.1155/2014/636123

**Published:** 2014-04-24

**Authors:** Irene Aprile, Marco Rabuffetti, Luca Padua, Enrica Di Sipio, Chiara Simbolotti, Maurizio Ferrarin

**Affiliations:** ^1^Don Carlo Gnocchi Foundation, SM Provvidenza Movement Laboratory, 00166 Rome, Italy; ^2^Don Carlo Gnocchi Foundation IRCCS, Biomedical Technology Department, 20148 Milan, Italy; ^3^Institute of Neurology of Catholic University, 00168 Rome, Italy

## Abstract

Advanced rehabilitation strategies of the upper limb in stroke patients focus on the recovery of the most important daily activities. In this study we analyzed quantitatively and qualitatively the motor strategies employed by stroke patients when reaching and drinking from a glass. We enrolled 6 hemiparetic poststroke patients and 6 healthy subjects. Motion analysis of the task proposed (reaching for the glass, bringing it to the mouth, and putting it back on the table) with the affected limb was performed. Clinical assessment using the Fugl-Meyer Assessment for Upper Extremity was also included. During the reaching for the glass the patients showed a reduced arm elongation and trunk axial rotation due to motor deficit. For this reason, as observed, they carried out compensatory strategies which included trunk forward displacement and head movements. These preliminary data should be considered to address rehabilitation treatment. Moreover, the kinematic analysis protocol developed might represent an outcome measure of upper limb rehabilitation processes.

## 1. Introduction

Stroke is the third leading cause of death in Western countries and contributes significantly to the incidence of long-term physical disabilities and handicaps [1]. Up to approximately 85% of stroke survivors experience hemiparesis, resulting in an impairment of an upper limb (UL) immediately after the stroke. Furthermore between 55% and 75% of survivors continue to experience limitations in UL function, which are associated with diminished health-related quality of life, even after 3 to 6 months [2–11]. A good sensorimotor recovery in stroke patients is considered as the capacity of the patient to perform movements in the same way as age-matched nondisabled subjects.

Therefore a good sensorimotor recovery means not only being able to do the movement (quantitative aspect of movement) but also (and more important) knowing how the movement is done (qualitative aspect of movement).

In UL a good sensorimotor recovery may be slower or more complex than in lower limbs. One explanation for poor recovery of arm function may be the greater emphasis placed on retraining gait capability in the effort to mobilize the patient as quickly as possible and to minimize costly hospital stays [12]. Moreover UL movements are also far less stereotypical and more complex than lower limb (LL) movements, involving a wider amount of coordinated multijoint movements, including head, neck, trunk, and shoulder to manipulate objects in the environment.

Clinical outcome scales meant to measure improvement mainly focus on task accomplishment and are often not qualitatively sensitive enough to discriminate improvement in how the task is performed. Instrumental movement analysis can provide more specific information about qualitative movement components and strategies, but this requires special equipment and training and is most applicable in research setting. Kinematics describes movements of the body through space and time, including linear and angular displacements, velocity, and acceleration, but without reference to the forces involved [13, 14]. Three-dimensional imaging techniques, including optoelectronic systems, can provide a quantitative assessment of movement, but protocols and models for UL analysis are not fully established [15–17].

The focus of stroke rehabilitation is to maximize functional motor ability, such as walking safely from one room to another, or turning a doorknob to open a door, in the limited time available for treatment.

Manual and UL tasks are also difficult to analyze: generally such tasks are not cyclic; they are characterized by a large number of degrees of freedom and, consequently, can be performed adopting different strategies or motor patterns. This accounts for the relatively small number of published studies on instrumented analysis of UL tasks [18], compared to the larger amount of studies on locomotor functions, universally known as Gait Analysis. Drinking from a glass is a very common and important daily life activity. This task is paradigmatic since it includes two relevant phases: a first open-chain movement (reaching for the glass) and a second movement (bringing the glass to the mouth) where a strict coordination among upper limb, trunk, and head movements is required, thus challenging the motor control system even in the presence of UL minimal disability. Interestingly the task of bringing a glass to the mouth in poststroke patients has been considered only in one previous paper reported by Murphy et al. [16]. In Murphy's study [16] patients with subacute and chronic stroke were considered (with a stroke latency from 6 to 64 months). It is known that a long latency from stroke increases the possibility to develop spasticity [19]: this might explain why three of Murphy's patients presented spasticity at the paretic upper limb. Actually, compensatory motor strategies employed by patients can be influenced by UL spasticity and can change over time, depending on when the stroke had actually occurred. Therefore, it is reasonable to expect that motor strategies of upper limb are different between subacute and chronic patients. Moreover, in Murphy's study compensatory movements of shoulder abduction and trunk forward displacement, but not of trunk axial rotation, were considered.

The aim of this study was to analyze, using motion analysis, the quantitative and qualitative UL motor strategies adopted by stroke patients, without spasticity, in reaching for a glass and bringing it to the mouth.

## 2. Materials and Methods

### 2.1. Sample

We enrolled 6 hemiparetic poststroke patients (mean age: 78 years; range: 64–84 years; 3 men, 3 women) admitted to our Inpatient Rehabilitation Department.

Inclusion criteria for stroke patients were a subacute status (from 1 month to 6 months after event) following a first unilateral cortical or subcortical stroke and ability to perform the task proposed (reaching for the glass, bringing it to the mouth, and putting it back on the table) with the affected limb.

Exclusion criteria were the presence of musculoskeletal or neurological problems that could affect the function of the arm (trauma, fracture, and peripheral neuropathies) and poststroke spasticity of the affected arm (Ashworth Scale Score ≥ 2).

Two of the 6 patients enrolled had suffered from hemorrhagic stroke, four from ischemic stroke; four had right hemiparesis and two left hemiparesis. The mean value of the Barthel Index was 50 and the mean value of the Fugl-Meyer Assessment for Upper Extremity was 44.

Six healthy subjects (mean age: 64.5 years; range: 52–74 years; 6 women) were used as a control group.

All participants gave written consent to participate in the study, which was approved by our Ethical Committee.

### 2.2. Clinical Evaluation

The UL motor function was clinically assessed with the Fugl-Meyer Assessment for Upper Extremity (FMA-UE, scale 0–66) [20, 21]. FMA is an efficient and reliable tool useful in monitoring the progress of patients and to analyze comparatively the effectiveness of different therapeutic interventions [22]. Stroke patients included in this study had a moderate arm impairment (FMA-UE scores between 34 and 57; mean: 44.2; SD: 7.7).

The disability was assessed using Barthel Index (BI). It provides a measure of ability, measuring what an individual “can do.” The BI ranges from 0 (dependence) to 100 (independence). It is the most widely used measure to assess functional status, having great validity, reliability, and sensitivity. Our sample had a moderate disability (BI values between 34 and 58; mean: 49.5; SD: 9.3).

Clinical data of the stroke patients are provided in Table 1.

### 2.3. Motion Analysis and Motor Task Phases' Identification

**Figure 1:**
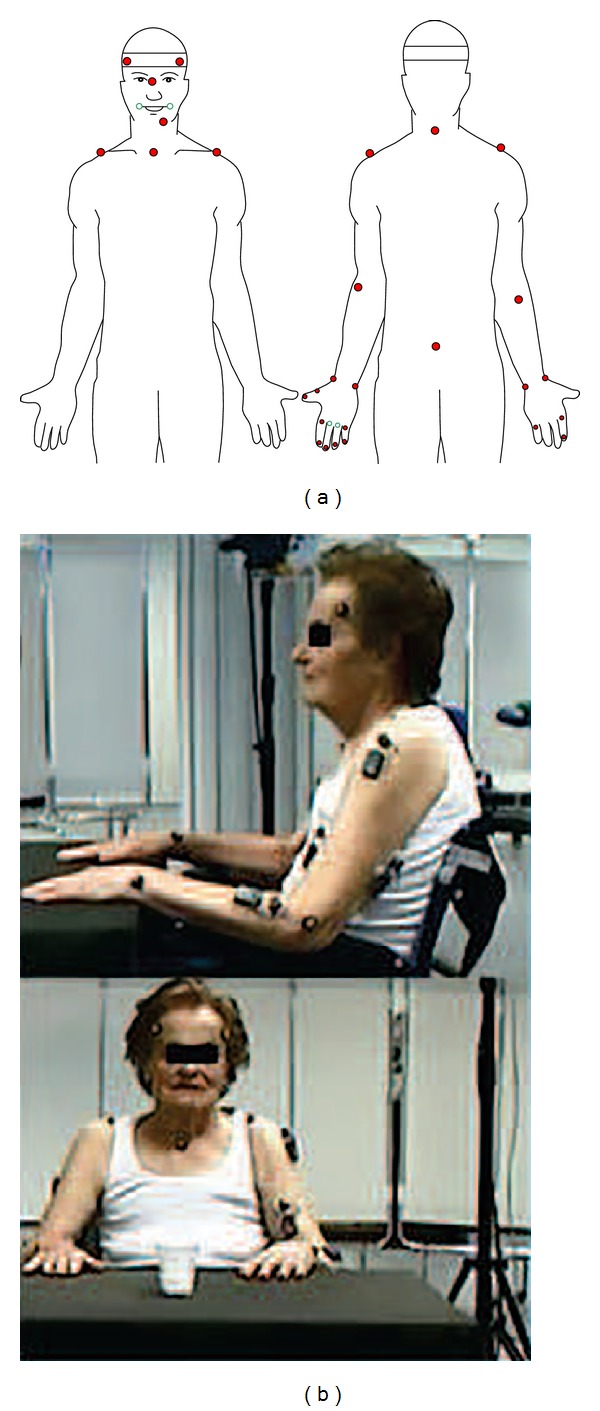
Markers positioning on the subjects and figure of our patient inside the research setting.

The task performance was measured by a SMART motion capture optoelectronic system, able to automatically record 3D trajectories of passive markers by means of stereophotogrammetric methods (BTS S.p.A., Milano, Italy). The experimental protocol included 30 markers on the body (see Figure 1 for detailed listing of markers' positions). The markers had a diameter of 6 mm, except for those placed on the trunk which had a 10 mm diameter (Figure 1). The SMART optoelectronic system was equipped with 8 cameras, working in infrared range and equipped with CCD sensors and appropriate optical filters; whose sampling frequency was 60 Hz (low pass filtered with five point triangular smooth).

The analyzed drinking task required the subject to reach for the glass with his/her affected limb, for subjects belonging to the stoke group, or with the preferred limb, for the control group. The subject was initially seated, with both hands placed on the table and the glass placed at 400 mm from the edge of the table aligned with the subject sagittal plane. The onset of the task (T1) was marked by any motion of the subject body, in particular the hand displacement motion with trunk fixed or the trunk movement with hand still resting on the table. The reaching for the glass with the hand (T2) marked the end of reaching phase and the beginning of bringing-to-mouth phase. The contact of the glass with the lips with a proper glass inclination of approximately 45° (T3) marked the end of bringing-to-mouth and the beginning of drinking phase. The end of drinking (T4) was identified similarly to the previous event T3 and the repositioning of the glass on the table (T5) (approximately in its initial position) concluded the task. Therefore, the identified task phases were reaching for the glass (RG, from T1 to T2), bringing the glass to the mouth (BGM, from T2 to T3), drinking from it (DG, from T3 to T4), and putting the glass back on the table (TBGT, from T4 to T5).

**Figure 2:**
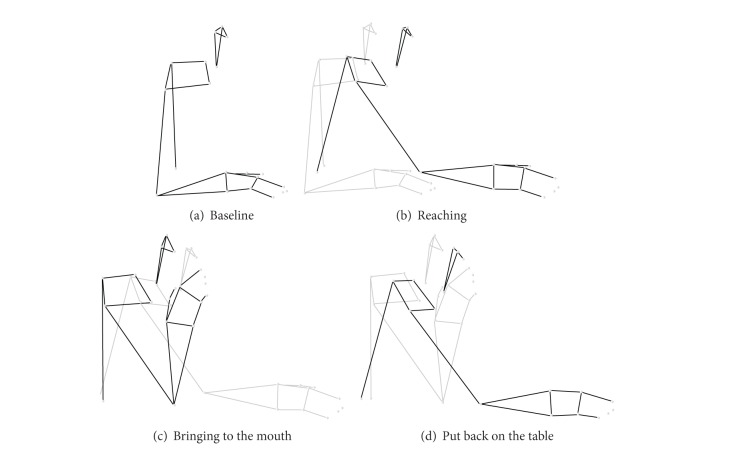
Motor task; (a) baseline; (b) reaching; (c) bringing to the mouth; (d) put back on the table.

Figure 2 shows an example of stick diagrams defining the phases of the motor task, in particular the baseline (a), the reaching for the glass (b), the bringing the glass to the mouth (c), and the putting the glass back on the table (d).

After the patient had practiced the task a few times (2-3 times), we registered three repetitions of the task that were then used for data analysis. Patients were instructed to perform the task at a comfortable self-paced speed after the examiner had announced, “you can start now.”

### 2.4. Quantitative Indexes

The measured markers' trajectories allowed us to compute several kinematic variables, including anatomical landmarks displacements, joints' angles, and body segments' orientations. Those variables supported the computation of scalar indexes concerning phases durations, joint ROM, and relative contribution of specific anatomical subparts to the whole body movement strategy.

In the forward direction, given the forward displacements of the end-effector, the grasping hand, and the forward movement of the proximal points, such as C7 and the shoulder (i.e., the acromion), it is possible to compute specific contribution, expressed in percentage, of the total hand displacement while reaching:

- *arm elongation* (AE), relative contribution to reaching (%) as the difference between hand and shoulder forward displacements relative to hand forward displacement;
- *trunk forward inclination* (TF), relative contribution to reaching (%) as the percentage ratio between C7 forward displacement and hand forward displacement;
- *trunk axial rotation* (TA), relative contribution to reaching (%) as the difference between shoulder and C7 forward displacements relative to hand forward displacement.

It is worth noting that, in the implemented model, the sum of AE, TF, and TA accounts for 100% of contributions to hand forward displacement in reaching.

Moreover the range of motion (ROM) of the elbow during reaching for the glass was evaluated.

Another index consisted of the mouth forward displacement during specific phases, that is, the displacement of a marker positioned on the head close to the mouth. This parameter while being largely determined by trunk inclination may be influenced also by neck movements. Given the mouth position (MP) at T1, T2, and T3 events, the following indexes are identified:

- MD_12_ = MP (T2) – MP (T1), mouth displacement in mm during reaching;
- MD_23_ = MP (T3) – MP (T2), mouth displacement in mm during bringing-to-mouth;
- MD_13_ = MP (T3) – MP (T1), overall mouth displacement in mm during reaching and bringing to mouth.

Arm contribution was evaluated also in bringing the glass to the mouth and putting it back on the table phases.

Smoothness of movement was quantified by computing the number of movement units (NMUs) during reaching for the glass and bringing the glass to the mouth. The number of movement units (NMU) was defined as the number of hand velocity peaks occurring above a threshold speed of 50 mm/s in the two initial phases (reaching for the glass and bringing the glass to the mouth).

In the present study we have focused on the quantitative indexes of three phases involving upper limb displacements: reaching for the glass (RG), bringing the glass to the mouth (BGM), and putting the glass back on the table (TBGT).

### 2.5. Statistical Analysis

All statistical analyzes were performed using the STATSOFT (Tulsa, OK, USA) package. Due to the small sample size, nonparametric analyses were performed. We used the Mann-Whitney *U* test for the comparison between two groups (stroke group and control group). Regarding the quantitative motion analysis indexes, for statistical calculations, the mean value of 3 trials was used for each participant.

## 3. Results and Discussion

Table 1 shows the clinical data of stroke patients.

**Figure 3:**
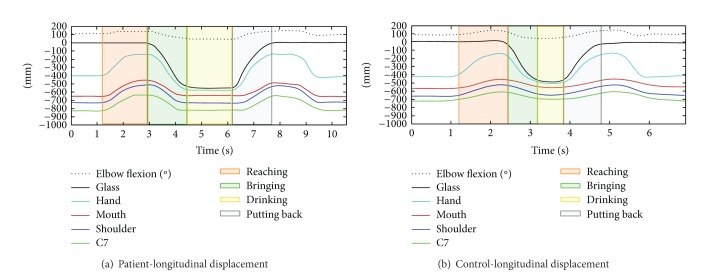
Longitudinal displacements during the whole task in a stroke patient (a) and in a healthy subject (b).

Figure 3 shows the longitudinal displacements during the task in a stroke patient Figure 3(a) and in a healthy subject Figure 3(b), respectively, of the glass (black line), hand (turquoise line), mouth (red line), shoulder (blue line), and C7 (green line). The dotted line represents the flexion/extension angular displacement of the elbow joint.

The colored panels have been used to highlight the movement phases, which are (i) reaching for the glass (red); (ii) bringing to the mouth (green); (iii) drinking (yellow); and (iv) putting the glass back on the table (grey).

It can be noticed that, in the shown example, the patient took about 7.5 seconds to complete the task, while the healthy subject needed less than 5 seconds.

**Figure 4:**
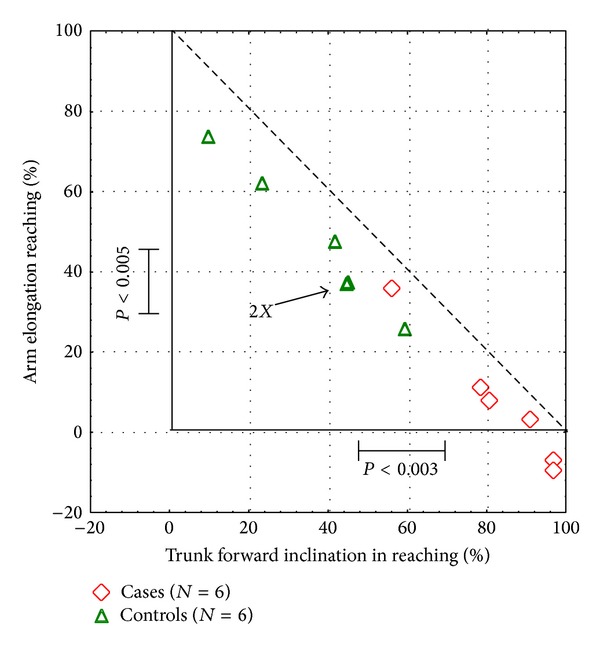
The contribution of the arm elongation and trunk forward inclination in the reaching phase in the two groups (stroke and control group).

**Figure 5:**
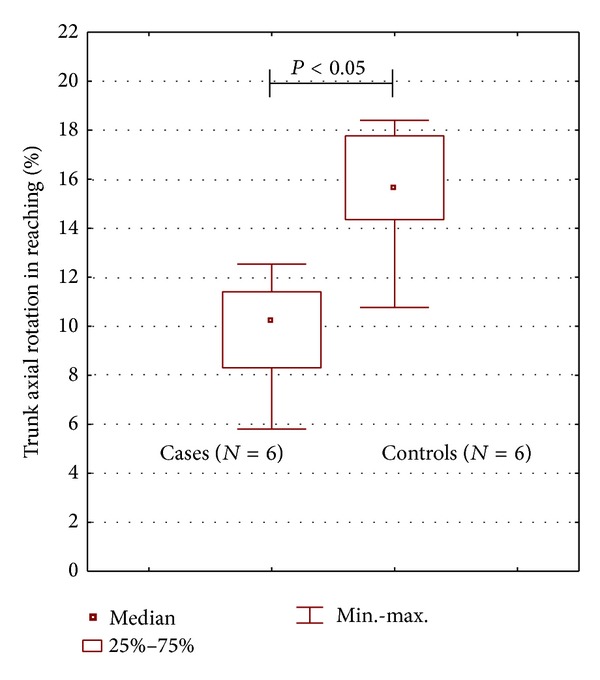
The comparison of the trunk axial rotation in the reaching phase in the two groups (stroke and control group).

In Table 2 the comparison between groups (stroke and control group) of the quantitative indexes relative to the phases of the task is reported. In the reaching phase, a significant reduction of the arm elongation (*P* < 0.005, Figure 4), the elbow ROM (*P* < 0.003), and the trunk axial rotation (*P* < 0.003, see Figure 5) was found in stroke patients compared to the control group. In the same phase, a significant increase of the duration (*P* < 0.003), the forward inclination of the trunk (*P* < 0.003, Figure 4), and the mouth forward displacement (*P* < 0.003) was found. A significant increase of duration was found also in the bringing and putting back phases (both *P* < 0.003) in patients in comparison with healthy subjects.

**Figure 6:**
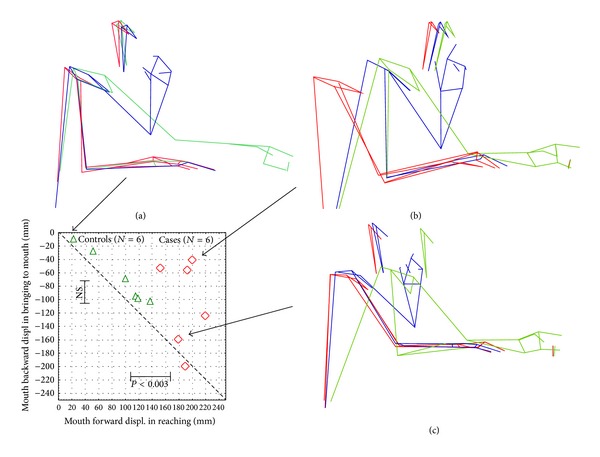
Head-trunk and arm motor strategy during the reaching for the glass and bringing it to the mouth in a healthy subject (a) and in 2 stroke patients ((b) and (c)). The graphic shows the mouth displacement in the bringing phase and the mouth displacement in the reaching phase in the sample.

In the graph of Figure 6, the mouth displacement in the bringing phase is plotted versus the mouth displacement in the reaching phase in patients and healthy subjects. Moreover the stick diagrams at the baseline, ending of reaching, and ending of bringing-to-mouth phases are reported for one healthy subject and two representative stroke patients, showing the different motor strategies observed during the task. It can be noticed that, during the reaching phase, the healthy subject in Figure 6(a) showed a wide elongation of the arm with a minimal forward movement of the trunk associated also with an axial rotation of the trunk. Concerning mouth movement of the healthy subjects, we observed (see graph) that the forward displacement in reaching—when present—is comparable to the backward displacement in the bringing phase. On the contrary two different strategies were observed in the patients. The first strategy in Figure 6(b) consisted of an important increase in the forward displacement of the trunk and mouth in the reaching phase but with a reduced arm elongation and without a comparable backward displacement of the trunk and the mouth in the bringing-to-mouth phase. In this case, the patient brings the glass to the mouth remaining in a “forward displaced head and trunk posture.” The second strategy in Figure 6(c), conversely, is characterized by an important increase in the forward displacement of the trunk and mouth in the reaching phase, with a reduced arm elongation, but with an equal backward displacement of the trunk and mouth in the bringing phase.

**Figure 7:**
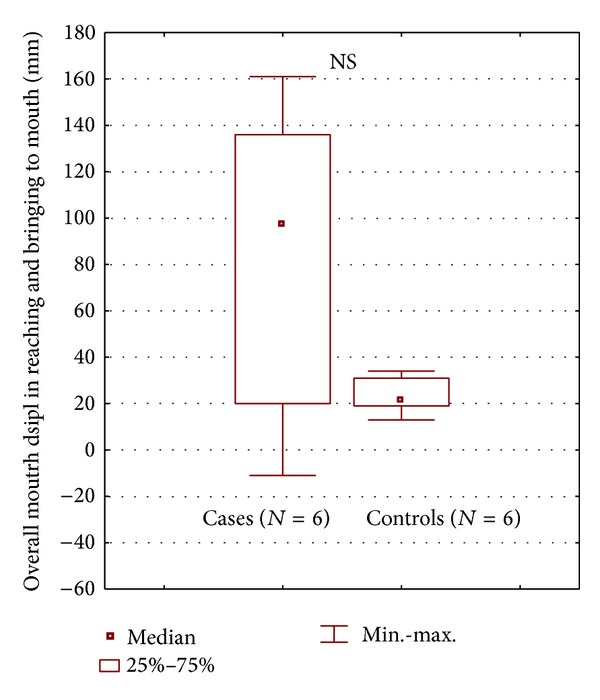
Comparison of the overall mouth displacement in reaching and bringing to the mouth in the two groups (stroke and control group).

Figure 7 reports the overall mouth displacement during reaching and bringing phases in stroke patients and control group.

The NMU showed statistical differences between the two groups; it ranged from 2 to 3 in healthy participants and from 2 to 26 in stroke patients (*P* < 0.0005).

## 4. Conclusions

Advanced rehabilitation strategies of the UL in stroke patients should focus on the recovery of the most important daily activities. Usually, with the emphasis on task accomplishment, little attention is given to qualitative aspects of movement and it is hard to distinguish between “primary recovery” and “secondary compensatory strategies” at the level of the basic motor patterns employed. On the other hand, such distinction is valuable given that, while primary recovery could allow patients to perform several motor tasks, compensatory strategies may not generalize to a wide array of tasks. Therefore any method, like the one here proposed, capable of objectifying this distinction is of major importance in the validation of new rehabilitation approaches.

The clinicians should pay attention not only to the ability of the patient to perform a task (which is often possible only using compensatory strategies) but also to how the patient performs the task. In this study we analyzed quantitatively and qualitatively the motor strategies employed by stroke patients when reaching and drinking from a glass.

During the reaching for a glass the patients showed a reduced arm elongation and trunk axial rotation due to motor deficit. For this reason, as observed, they carried out compensatory strategies which included trunk forward displacement and head movements. In the overall mouth displacement during reaching and bringing phases we observed a trend towards a more advanced mouth position with respect to the initial position in stroke patients than in controls, without statistical significance probably due to the small sample size. However we also observed a higher variability of this parameter in stroke patients probably due to the differences among individual strategies.

In a previous study that Murphy et al. investigated, the authors found that, in stroke patients affected 3 or more months earlier, variables describing movement time, smoothness, and velocity (resp., total movement time, number of movement units, and peak angular velocity of elbow) discriminated best between healthy subjects and patients with stroke (as well as patients with moderate versus mild arm impairment). They also observed that variables describing compensatory movement of the trunk and arm (trunk forward displacement while reaching for the glass and higher shoulder abduction while drinking) discriminated between patients with moderate and mild stroke impairment [16]. Our study shares Murphy's interest for a specific task but we provided a different set of variables.

We considered the reaching task as a global movement of hand forward displacement, of which we quantified the different contributing components: arm elongation, trunk forward inclination, and trunk axial rotation. Differently from Murphy our approach permitted us to show that trunk axial rotation of patients was significantly lower than that of controls. The reduction showed by stroke patients in arm elongation (as also shown by Murphy) and in trunk axial rotation (as only shown in the present study) demonstrated that the trunk forward inclination was of the utmost importance when patients had to reach for the glass.

It is worth noting that, although smaller, our sample of patients was more homogeneous than that of Murphy, who included both subacute and chronic patients, some of whom with spasticity at upper limb.

As regards the smoothness of movement, quantified by NMUs, it has appeared to be a very important parameter to discriminate movement quality between stroke patients and healthy subjects, as previously observed by Murphy.

These preliminary data need to be confirmed in further studies on a large population of patients with different severity of stroke. The kinematic analysis protocol that we developed and used in the current study might represent an outcome measure of UL rehabilitation processes, also in patients with UL disability due to different diseases/traumas (not only after stroke).

## Figures and Tables

**Table 1:** Clinical data of the sample.

Case	Gender	Age	Latency from stroke	Kind of stroke	Affected arm	Disability and Performance scales	
						Barthel Index (0–100)	Fugl-Meyer Scale (0–66)
Case CR	Male	76	3 months	Hemorrhagic	Right	52	46
Case LG	Male	83	6 months	Ischemic	Left	57	38
Case SF	Female	80	2 months	Ischemic	Right	58	42
Case SFl	Female	64	1 month	Ischemic	Right	56	53
Case CE	Female	81	3 months	Hemorrhagic	Left	34	48
Case SA	Male	84	5 months	Ischemic	Right	41	34

**Table 2:** Quantitative Indexes related to the phases of the task: comparison between patients' and controls' group.

Phase	Index	Patients' median	Patients' range	Controls' median	Controls' range	Mann-Whitney *P* value
Reaching for the glass	Duration (s)	3.09	1.62–3.54	1.24	1.09–1.41	**<0.003**
	Arm elongation (%)	5.63	−9.27–35.83	49.21	34.10–69.50	**<0.005**
	Elbow ROM (°)	10.10	3.37–30.70	48.40	34.20–66.80	**<0.003**
	Trunk forward Inclination (%)	85.77	55.90–96.70	37.84	14.67–52.53	**<0.003**
	Trunk axial rotation (%)	10.20	5.80–12.53	14.15	9.25–17.37	**<0.05**
	Mouth forward displacement (mm)	190.33	152.40–218.75	97.07	31.63–129.75	**<0.003**
	Number of movement units of reaching	5	1–9	1	1-2	**<0.0002**

Bringing the glass to the mouth	Duration (s)	2.59	1.68–4.61	1.46	1.11–1.64	**<0.003**
	Arm contribution (%)	66.37	29.00–72.73	65.85	60.00–82.03	NS
	Mouth backward displacement (mm)	−90.13	−199.47–−39.00	−80.95	−104.35–−16.77	NS
	Number of movement units of bringing	4	1–17	1	1-2	**<0.003**

Putting the glass back on the table	Duration (s)	2.22	1.85–3.89	1.44	1.24–1.52	**<0.003**
	Arm contribution (%)	60.73	29.77–76.77	65.11	61.37–80.57	NS

Number of movement units of reaching and bringing		9.5	2–26	2	2-3	**<0.0005**
